# Case Report: Two cases of long-term survival in advanced pancreatic cancer patients following treatment with *KRAS* G12C inhibitors

**DOI:** 10.3389/fonc.2025.1691760

**Published:** 2025-10-28

**Authors:** Saeed Aslani, Owen McKay, Lara Lipton, Vinod Ganju, Daniel Croagh

**Affiliations:** ^1^ Department of Surgery, School of Clinical Sciences, Monash University, Clayton, VIC, Australia; ^2^ Department of Gastroenterology and Hepatology, Monash Health, Melbourne, VIC, Australia; ^3^ Department of Medical Oncology, Western Health, VIC, Australia; ^4^ Oncology Department, Peninsula And Southeast Oncology, Frankston, VIC, Australia

**Keywords:** pancreatic ductal adenocarcinoma, KRAS, G12C inhibitor, chemotherapy, case report

## Abstract

Pancreatic ductal adenocarcinoma (PDAC) is a highly aggressive malignancy with a dismal prognosis. Molecular profiling to improve the diagnosis and management of PDAC holds promise for informing more targeted therapies such as Kirsten rat sarcoma viral oncogene homologue (KRAS) G12C inhibition to deliver better outcomes for these patients. In this report, we present two patients with advanced PDAC with *KRAS* G12C mutations who achieved remarkable disease control and prolonged survival following treatment with the *KRAS* G12C inhibitor D1553 (garsorasib). Case 1, a 74-year-old woman with recurrent PDAC post-surgical resection and chemotherapy, exhibited a significant biochemical and radiologic response upon initiation of targeted therapy. Case 2, a 75-year-old woman initially treated with Folinic acid, fluorouracil, irinotecan and oxaliplatin (FOLFIRINOX) and stereotactic body radiotherapy (SBRT), demonstrated sustained disease stability for over three years on *KRAS* G12C inhibitor therapy. Both patients maintained excellent performance status with minimal treatment-related toxicity. These cases underscore the potential of KRAS-directed therapies in PDAC and illustrate the importance of molecular profiling in identifying eligible patients. The findings support further investigation into the durability of *KRAS* G12C inhibition, resistance mechanisms, and combination treatment strategies to optimize patient outcomes.

## Introduction

Pancreatic ductal adenocarcinoma (PDAC) is the most common type of pancreatic cancer, accounting for about 90% of all cases. PDAC ranks as the seventh most common cause of cancer-related mortality worldwide with a five-year survival rate below 10%. However, projections indicate that by 2030, it will rise to become the second leading cause of cancer deaths globally (1). In 2025, an estimated 67,440 Americans are expected to be diagnosed with PDAC, with approximately 51,980 anticipated deaths (2). The incidence rates are approximately four times higher in countries with a higher Human Development Index (HDI) compared to those with a lower HDI with the highest rates of occurrence are observed in Europe, North America, and Australia/New Zealand (1). Currently, over 80% of individuals diagnosed with PDAC have either locally advanced or metastatic disease at the time of presentation. In such cases, systemic chemotherapy is the primary therapeutic approach. Although there have been gradual improvements in treatment strategies, the overall outlook for patients remains bleak, with a median five-year survival rate lingering at a mere 10% (3).

Genetic alterations in KRAS are detected in nearly 90% of pancreatic ductal adenocarcinomas, the most common form of pancreatic malignancy (4). The *KRAS* G12C variant (characterized by the substitution of glycine with cysteine at codon 12) is identified in roughly 1–2% of affected individuals (5). Sotorasib, a targeted small-molecule inhibitor, selectively and irreversibly binds to *KRAS* G12C, blocking its oncogenic activity. The U.S. Food and Drug Administration granted expedited approval for sotorasib in the treatment of patients with *KRAS* G12C mutated non–small-cell lung cancer (NSCLC) who have undergone at least one prior systemic therapy (6). D-1553 (garsorasib), a potent and selective inhibitor of *KRAS* G12C mutation, represented a promising therapeutic agent for treating NSCLC patients with this mutation (7). Additionally, a single-group phase 1–2 trial indicated that in individuals with advanced PDAC harboring the *KRAS* G12C mutation who had undergone prior systemic therapy, sotorasib demonstrated therapeutic efficacy against the malignancy while maintaining a tolerable safety profile. The treatment was associated with a median overall survival (OS) of 6.9 months and a progression-free survival (PFS) of 4.0 months (8).

The application of *KRAS* G12C inhibitors in PDAC is still being evaluated, but early clinical data suggests therapeutic benefits for selecting patients. In this report, we describe two exceptional cases of advanced PDAC patients with *KRAS* G12C mutations who achieved long-term disease control following treatment with the investigational KRAS *G12C* inhibitor D1553. These cases highlight the potential of targeted therapy informed by molecular profiling to potentially extend survival and improve quality of life in PDAC. Furthermore, they underscore the importance of continued research into combination therapies and resistance mechanisms to optimize treatment efficacy and patient outcomes.

## Methods

Clinical and molecular information for the patients were sourced from the Victorian Pancreatic Cancer Biobank and Endoscopic Ultrasound Molecular Evaluation of Pancreatic Cancer (EU-ME-PC) trial (ACTRN12620000762954) (9), following approval from the local institutional ethics committees (HREC/15/MonH/117 and HREC/61006/MonH-2020-200407). Prior to inclusion, patients gave explicit written consent for the use of anonymized clinical and molecular data, as well as deidentified images, in research and publication. KRAS mutations were initially identified using the KRAS StripAssay (ViennaLab). To further characterize the tumors, comprehensive genomic profiling was conducted using the TruSight™ Oncology 500 (TSO-500) panel, which requires a minimum tumor cellularity of approximately 10-20% to ensure reliable variant detection (although variant allele frequencies as low as 1% were accepted) as part of the EU ME PC trial (9).

## Case description

### Case 1

A 74-year-old woman presented with progressive abdominal and back pain, along with significant unintentional weight loss over several months in July 2018. Her past medical history included osteoarthritis (OA) with bilateral hip replacements.

**Figure 1:**
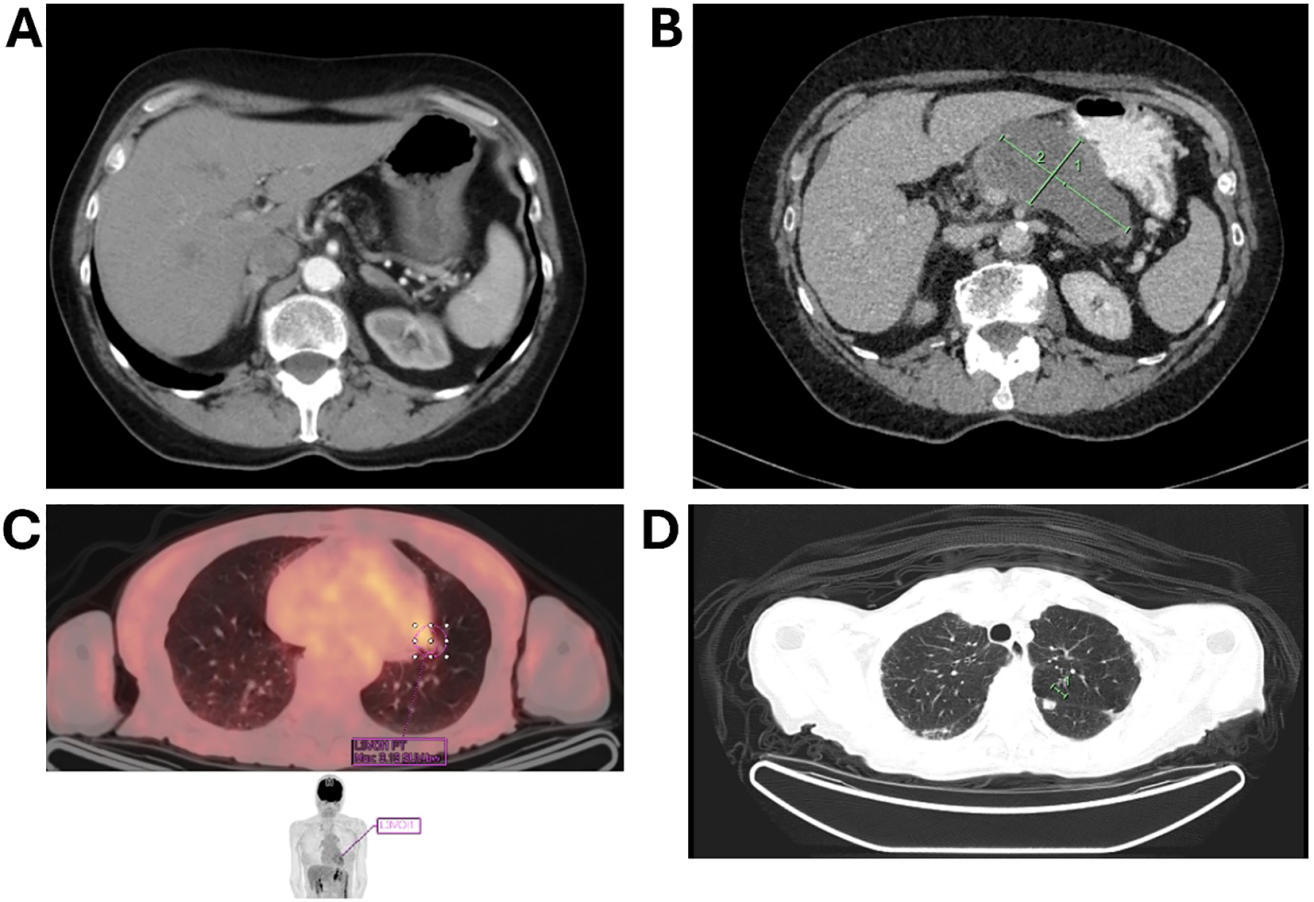
CT imaging comparison demonstrating an 8.7 × 4.5 × 5.3 cm pancreatic mass. **(A)** earlier scan; **(B)** a scan after 8 years, illustrating significant morphological changes over 8 years; **(C)** imaging of a solitary lung lesion indicative of early metastatic spread; **(D)** CT scan demonstrating a second lung lesion treated with radiotherapy.

A CT scan revealed an 8.7 × 4.5 × 5.3 cm pancreatic mass. A comparative analysis of prior imaging (**Figure 1A**) and the most recent scan (**Figure 1B**) showed significant morphological changes over an 8-year period. Notably, serum CA 19–9 was significantly elevated at 5542 U/m, but liver function tests were normal.

Subsequent MRI revealed a heterogeneous mass within the pancreatic body and tail without notable vascularity or enhancement associated with portal venous stenosis with splenic vein occlusion. Endoscopic ultrasound showed a 15 cm hypodense mass; FNA cytology was inconclusive due to extensive necrosis. A separate pass was taken and frozen for biobanking with the plan for subsequent genomic profiling.

The patient was diagnosed with borderline resectable pancreatic cancer and was referred for pseudoneoadjuvant chemotherapy. Treatment with gemcitabine and nab-paclitaxel resulted in a marked reduction in tumor size and CA 19–9 level, which fell to 58 IU/mL (**Figure 1C**). However, chemotherapy was discontinued after 2.5 cycles due to severe skin toxicity.

The patient then underwent surgical resection with en-bloc distal pancreatectomy, splenectomy, and near-total gastrectomy, along with a circumferential portal vein resection. The final pathology report confirmed pT3N0 disease with invasion into the gastric muscularis propria. All surgical margins were negative, and no lymphovascular or perineural invasion was detected. A *KRAS* StripAssay revealed a *KRAS* G12C mutation.

**Figure 2:**
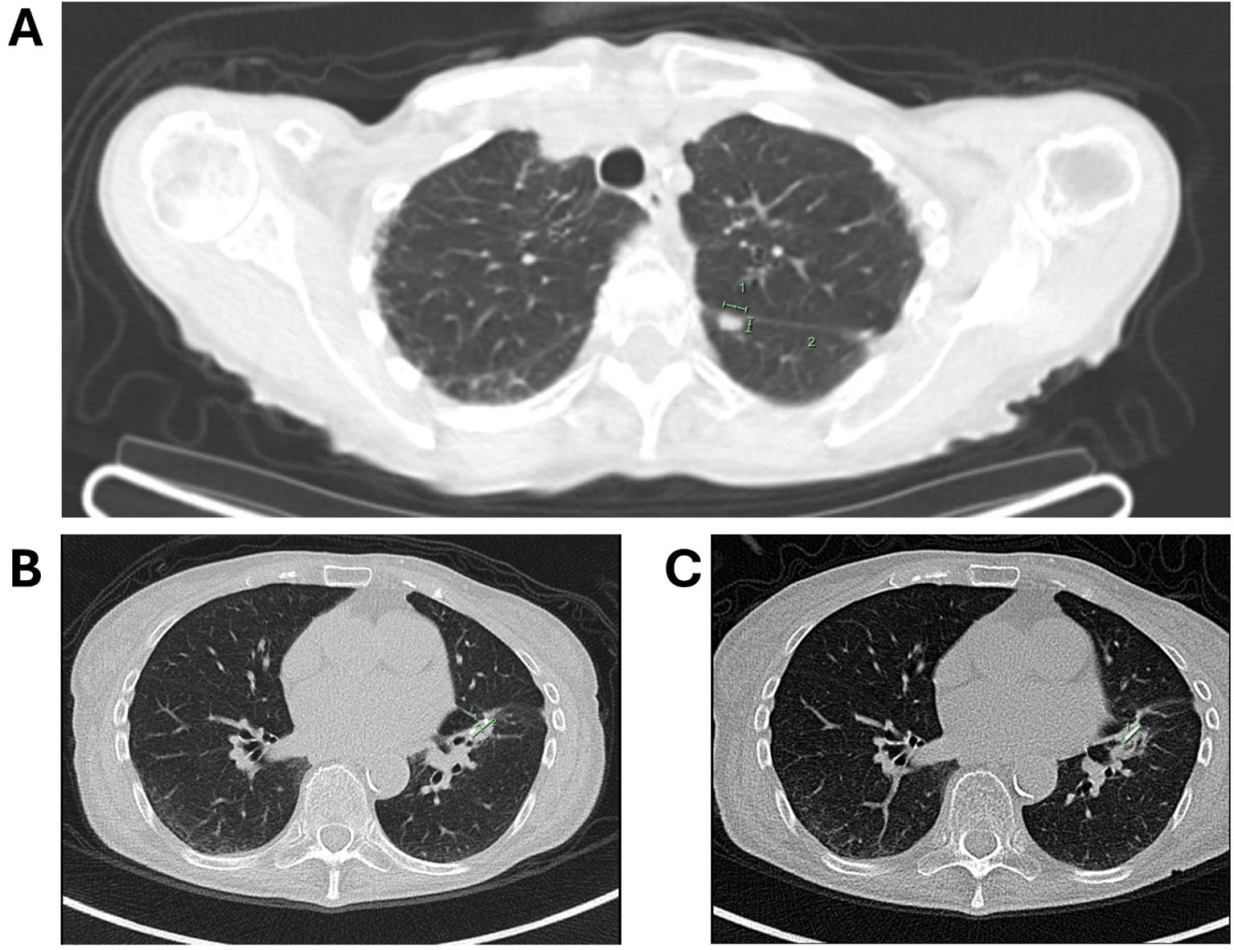
**(A)** Follow-up imaging showing a good response to radiotherapy for the second lung lesion; **(B)** imaging depicting the recurrence of the first lung lesion; **(C)** subsequent imaging showing recurrence of the first lung lesion with a favorable response to radiotherapy.

During routine follow-up, approximately 12 months after surgery (around 18 months from initial diagnosis), serial imaging and biomarker assessments showed disease progression. A solitary lung lesion was identified and successfully resected which was confirmed to be metastatic pancreatic cancer (**Figure 1C**). Later, a second pulmonary lesion was detected and treated with radiotherapy (**Figure 1D**), with subsequent imaging demonstrating a positive therapeutic response (**Figure 2A**). Despite these interventions, recurrence of the initial lung lesion occurred (**Figure 2B**), necessitating repeat radiotherapy, which again yielded a favorable response (**Figure 2C**). Eventually, new PET-avid pulmonary lesions were detected, coinciding with a rise in CA 19–9 levels, suggesting further disease progression.

She was enrolled in the EU ME PC study and TSO-500 panel identified the previously known *KRAS* G12C mutation. Additional findings included a low Tumour Mutation Burden (TMB) at 6.3 mutations per megabase (mut/Mb), and the absence of Microsatellite Instability (MSI) with only 0.9% unstable sites. The KRAS G12 Variant Allele Frequency (VAF) was 12.6%.

**Figure 3:**
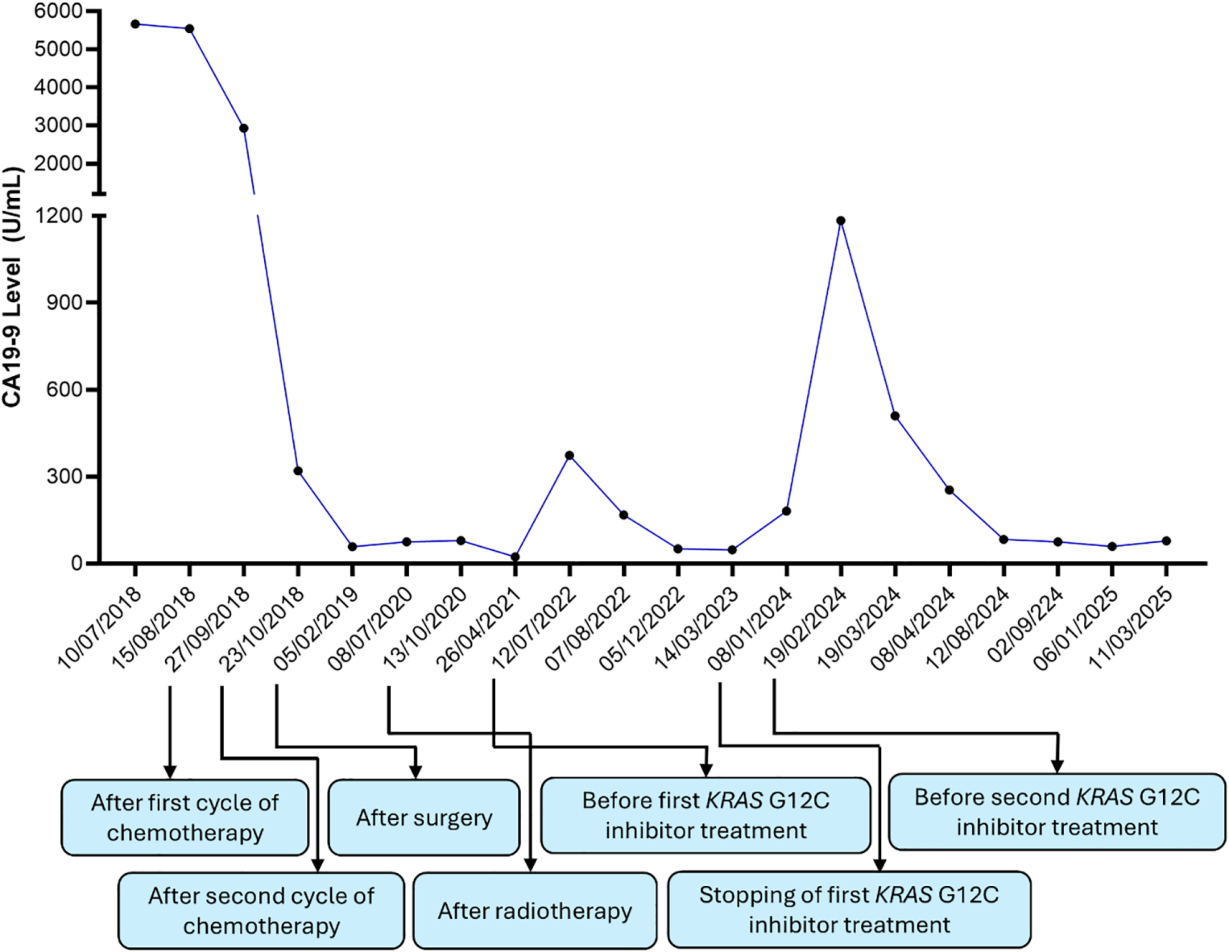
Level of CA19–9 in Case 1 over the course of disease and various interventions.

Given these findings, the patient was enrolled in a clinical trial investigating a *KRAS* G12C inhibitor, D1553 on 8 June 2022 (NCT04585035). Following the initiation of *KRAS* G12C inhibitor therapy, the patient exhibited a significant biochemical response, with a fall in CA 19–9 levels and a complete metabolic response on PET scan. The treatment was associated with moderate diarrhea (intermittent, grade 1-2) which was controlled with loperamide. The response was durable, lasting almost 16 months before a gradual rise in CA 19-9, suggesting resistance. She then commenced on a second KRAS G12C inhibitor, RMC-6291, also known as elironrasib as part of a clinical trial (NCT05462717), with a further complete metabolic response and return of CA 19–9 to near normal (**Figure 3**). The patient remains well for 7 years following the initial diagnosis.

### Case 2

A 75‐year‐old (original age of patient at start) female who had no significant past medical history and first presented with vague abdominal pain and nausea in late 2019. Imaging revealed a locally advanced pancreatic mass in the uncinate process with arterial and venous encasement but no distant metastases. EUS-FNA confirmed PDAC. She received eight cycles of modified FOLFIRINOX with excellent radiological and biochemical response, followed by stereotactic body radiotherapy (SBRT).

Molecular testing of the formalin-fixed EUS-FNA biopsy using a KRAS StripAssay previously identified a KRAS G12C mutation. Comprehensive molecular profiling was then attempted on the formalin-fixed EUS-FNB specimen with the TSO-500 panel; however, this was unsuccessful. Her CA19–9 level subsequently rose to 103, and repeat CT and PET imaging in May 2022 demonstrated persistent avidity in the uncinate process tumor without evidence of metastases. She was enrolled in the trial investigating D1553–101 as described above (NCT04585035). Over more than three years on therapy, she has maintained sustained disease control, with serial imaging showing partial response (primary lesion reduced from ~15 mm to 10 mm) and a persistent normalization of her CA19–9 level. Regular assessments by the oncology team have confirmed that her disease remains controlled, reflecting a favorable biological response to targeted therapy and she has maintained an excellent performance status throughout her treatment. The targeted therapy has been well tolerated overall, with only minor adverse effects reported. She experiences mild gastrointestinal symptoms (characterized by fluctuating bowel habits and low-grade abdominal cramping) which have been managed effectively with supportive measures such as pancreatic enzyme supplementation (Creon) and, when necessary, dose adjustments of D1553-101 (for instance, a temporary reduction to 400 mg twice daily was implemented to alleviate symptoms).

Just over four years after her PDAC diagnosis, she developed right-sided colon cancer [T3N0, mismatch repair (MMR) deficient *B-Raf proto-oncogene, serine/threonine kinase* (*BRAF*) positive tumor consistent with a colorectal primary] treated with laparoscopic right hemicolectomy without adjuvant therapy. She is now almost 6 years from her initial PDAC diagnosis, with high overall quality of life and normal daily activity.

## Discussion

The cases described here exemplify the transformative potential of targeted therapy in PDAC, particularly in the subset of patients harboring the *KRAS* G12C mutation. Historically, PDAC has been associated with poor prognosis, limited treatment options, and resistance to conventional chemotherapy (10). Both of these patients had *KRAS* testing as part of screening for potential inclusion in a trial of anti- Epidermal growth factor receptor (EGFR) monoclonal antibody, panitumumab, in *KRAS* wild-type pancreatic cancer (11). Although they were not candidates for that trial with the advent of *KRAS* G12C inhibitors, they became eligible for an exciting new therapeutic approach for a mutation that long considered undruggable (12). However, there is still only a handful of clinical trials reporting the efficacy of *KRAS* G12C inhibitors in improving survival in PDAC (8, 13–16).

*KRAS* mutations occur in over 90% of pancreatic ductal adenocarcinomas (PDAC), with G12D being the most common variant. *KRAS* G12C, while less frequent, offers the opportunity for targeted intervention due to the development of covalent inhibitors that irreversibly bind to the mutant cysteine residue, disrupting oncogenic signaling (17). There is limited data on the efficacy of *KRAS* G12C inhibitors in improving OS in PDAC subjects. A recent study revealed that the median PFS was 4.0 months (95% CI, 2.8 to 5.6), and the median OS was 6.9 months (95% CI, 5.0 to 9.1) in PDAC patients receiving Sotorasib (8). Since our cases demonstrated nearly 40 months of survival (as of July 2025) after treatment with D1553, they are considered exceptional. D1553, the investigational *KRAS* G12C inhibitor administered to both patients in this study, provided sustained clinical benefit, highlighting the therapeutic potential of targeting this pathway. It should be noted that a trial (NCT04585035) on the efficacy of D1553 on *KRAS* G12C harboring PDAC subjects is underway and will add to the potential therapeutic arsenal available for these patients.

Case 1 exhibited progressive disease despite multimodal interventions, including chemotherapy, extensive surgical resection, and metastasectomy and radiotherapy for pulmonary recurrences. Her genomic profile revealed a low TMB and stable MSI, suggesting limited responsiveness to immunotherapy (18). The identification of *KRAS* G12C allowed enrollment in a clinical trial, providing a molecularly guided treatment strategy. Within months of initiating therapy, a marked biochemical response was observed, as indicated by declining CA 19–9 levels. The durable disease control seen in the patient, who was previously refractory to a combination of local and systemic therapies, highlights the efficacy of *KRAS* G12C inhibition in a heavily pretreated patient.

Similarly, case 2 demonstrated an exceptional response following treatment with D1553. Her initial management with FOLFIRINOX and SBRT provided significant tumor reduction; however, the sustained benefit over a three-year period while on *KRAS* G12C inhibition is remarkable. The stable disease status, maintained tumor shrinkage, and minimal toxicity emphasize the potential of targeted therapies to not only improve survival but also maintain quality of life in patients with advanced PDAC. Her case further validates the role of *KRAS*-directed therapy as an effective long-term treatment strategy.

Unlike conventional chemotherapy, which often results in significant toxicity limiting long-term use, *KRAS* G12C inhibitors offer a more tolerable safety profile (8). Both patients remained active and independent, experiencing only mild gastrointestinal symptoms that were effectively managed with pancreatic enzyme supplementation (Creon) and dose adjustments. This is a critical consideration in PDAC management, where treatment-induced morbidity frequently diminishes quality of life.

In case 2, ongoing monitoring confirmed disease stability with no new metastatic lesions - a notable outcome given the historical progression pattern of PDAC. Case 1, despite prior pulmonary metastases, achieved prolonged disease control, further reinforcing the durability of *KRAS*-targeted inhibition. The ability to maintain stable disease over extended periods is particularly relevant given the limited options traditionally available for patients who progress on standard chemotherapy.

While the results observed in these cases are encouraging, several challenges remain in the broader application of *KRAS* G12C inhibitors. One of the primary concerns is the potential emergence of resistance mechanisms, which have been reported in other malignancies treated with *KRAS*-targeted agents (19). In our case, repeat biopsy was not performed at the time of resistance after 16 months on D1553 therapy; therefore, we were unable to directly characterize the molecular mechanisms underlying resistance. However, prior studies have identified several mechanisms of acquired resistance to KRAS G12C inhibitors. These include secondary mutations within KRAS itself (such as Y96D, R68S, and H95 substitutions) that impair drug binding, KRAS amplification leading to sustained signaling, and alterations in parallel or downstream pathways including RTK, PI3K, SHP2, and MAPK signaling (12). While our patient was subsequently enrolled in a second KRAS G12C inhibitor trial (RMC-6291), the precise resistance mechanism remains unknown. This highlights the importance of repeat biopsy and molecular profiling at progression to inform next-line therapeutic strategies.

Adaptive signaling pathways, secondary mutations, and compensatory oncogenic circuits may eventually limit the efficacy of monotherapy approaches. Future research should explore combination strategies, such as dual inhibition of *KRAS* and other key signaling pathways (such as SHP2, PI3K, or MEK), to enhance and prolong responses. Furthermore, *KRAS* mutations are not uniform in PDAC, and only a subset of patients harbors the G12C alteration. Expanding the therapeutic reach of targeted agents to include other *KRAS* variants, such as G12D and G12V, remains an active area of investigation (17). The development of pan-*KRAS* inhibitors and allele-specific agents will be crucial in broadening treatment applicability.

It should be noted that germline testing was not performed in these cases. Nonetheless, germline alterations in DNA damage repair genes such as *BRCA1/2*, *PALB2*, and *ATM*, as well as MMR deficiency, are clinically relevant in PDAC due to their therapeutic implications (20). Patients harboring *BRCA1/2* or *PALB2* mutations may benefit from platinum-based chemotherapy and Poly (ADP-ribose) polymerase (PARP) inhibitors (21), whereas those with MMR deficiency may be eligible for immune checkpoint inhibitor therapy (22). Notably, Case 2 later developed an MMR deficient colon cancer, although this appears to have been somatic rather than germline given the positive B-raf immunohistochemistry. Nevertheless, this highlights the importance of incorporating germline testing into the management of PDAC, both to guide targeted therapy and to inform genetic counseling for patients and families.

## Conclusion

These two cases signal a paradigm shift in the management of PDAC with the move toward more targeted therapy such as *KRAS* G12C inhibitors. The remarkable clinical responses, sustained disease control, and improved quality of life underscore the potential of molecularly driven therapies in an otherwise treatment-refractory malignancy. Nonetheless, we acknowledge that these cases may be subject to selection and confounding biases, and the prolonged survival could partly reflect unusually indolent tumor biology rather than solely the therapeutic effect of KRAS G12C inhibition. As case reports inherently highlight exceptional outcomes, the findings should be interpreted with caution until validated in prospective clinical trials. While further research is needed to optimize treatment sequencing, overcome resistance, and expand the spectrum of targetable *KRAS* mutations, these cases provide compelling evidence supporting the integration of targeted therapy into PDAC management. The success of *KRAS* G12C inhibition represents a significant step forward in addressing the unmet needs of this challenging disease, offering renewed hope to patients and clinicians alike.

## Data Availability

The original contributions presented in the study are included in the article/supplementary material, further inquiries can be directed to the corresponding author/s.
